# Hydrogel Microtumor Arrays to Evaluate Nanotherapeutics

**DOI:** 10.1002/adhm.202201696

**Published:** 2022-11-28

**Authors:** Yiling Liu, Stephanie Nemec, Chantal Kopecky, Martina H. Stenzel, Kristopher A. Kilian

**Affiliations:** ^1^ School of Chemistry The University of New South Wales Sydney NSW 2052 Australia; ^2^ Australian Centre for NanoMedicine Sydney NSW 2052 Australia; ^3^ School of Materials Science and Engineering The University of New South Wales Sydney NSW 2052 Australia; ^4^ Adult Cancer Program The University of New South Wales Sydney NSW 2052 Australia

**Keywords:** biomaterials, cancer stem cells, drug development, nanoparticles, tumor microenvironment

## Abstract

Nanoparticle drug formulations have many advantages for cancer therapy due to benefits in targeting selectivity, lack of systemic toxicity, and increased drug concentration in the tumor microenvironment after delivery. However, the promise of nanomedicine is limited by preclinical models that fail to accurately assess new drugs before entering human trials. In this work a new approach to testing nanomedicine using a microtumor array formed through hydrogel micropatterning is demonstrated. This technique allows partitioning of heterogeneous cell states within a geometric pattern—where boundary regions of curvature prime the stem cell‐like fraction—allowing to simultaneously probe drug uptake and efficacy in different cancer cell fractions with high reproducibility. Using melanoma cells of different metastatic potential, a relationship between stem fraction and nanoparticle uptake is discovered. Deformation cytometry reveals that the stem cell‐like population exhibits a more mechanically deformable cell membrane. Since the stem fraction in a tumor is implicated in drug resistance, recurrence, and metastasis, the findings suggest that nanoparticle drug formulations are well suited for targeting this dangerous cell population in cancer therapy.

## Graphical abstract

Hydrogel micropatterning is used to confine melanoma cells, leading to spatial segregation of stem cell‐like populations. Treatment with nanodrugs shows preferential uptake in stem fraction, which is due to increased membrane deformability. This finding suggests nanoparticle formulations of drugs may be well suited for targeting the stem‐like cells that are responsible to recurrence, resistance, and metastasis in cancer.

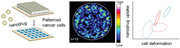

## 1 Introduction

Cancer is a leading cause of death and despite many years of intense research, effective therapies remain elusive, particularly in the treatment of advanced stages and disseminated disease. Nanoparticle‐based drugs are a promising method for treating cancer, where a high concentration of drug may be delivered to the tumor mass with considerably less off‐target effects compared to systemic treatment with small molecules.^[^ ^1^, ^2^ ^]^ Considering the vast potential for nanomedicine in cancer therapy, it is somewhat surprising that only 51 formulations have been approved for use over the last 30 years.^[^ ^3^, ^4^ ^]^ One reason for the slow pace of clinical translation is in part because of poor preclinical modeling, which has led to high variability in efficacy once the nanodrug reaches the clinical trial stage.^[^ ^5^, ^6^ ^]^ A significant problem with current preclinical models is their failure to replicate the heterogeneous environment in patient tumors, therefore resulting in an inaccurate assessment of toxicity and efficacy.

Preclinical drug testing often starts on a 2D platform, with a monolayer of adherent cells on polystyrene tissue culture plates. Unfortunately, the results of plate‐based cell culture assays frequently do not translate to patients, as these approaches fail to mirror the complexity of tumor tissue.^[^ ^7^ ^]^ In vivo models are a significant improvement with respect to complexity compared to monocultures. The current gold standard is the patient‐derived xenograft (PDX) model, which involves taking surgically resected tumor fragments from cancer patients, then directly transplanting into immunodeficient mice.^[^ ^8^, ^9^ ^]^ PDX models often maintain very similar cellular and histopathological structures of the parental tumors. However, they ultimately lose physiological similarity as the murine cells replace human stroma.^[^ ^10^, ^11^ ^]^ More recently, the use of in vitro organotypic systems appears as a viable compromise, with better 3D modeling properties compared to plate‐based formats, and a more accessible format compared to PDX. However, imaging 3D cell aggregates is time consuming and these models invariably require encapsulation in animal matrices, where batch‐to‐batch uniformity can cause issues in reproducibility.^[^ ^12^ ^]^ Another commonly adopted method is generating spheroids in a low‐adherent round bottom plate‐based format. While a uniform 3D tumor mimic can be created, this method is unable to expose cells to features of the extracellular matrix, a critical aspect for developing therapies on fibrotic and stroma‐rich cancers.

There is a clear need for preclinical methods where materials properties and cellular heterogeneity can be reproducibly controlled.

In this paper we use hydrogel micropatterning to control matrix stiffness and the geometry of pseudo‐3D cancer aggregates in a microarray format for the assessment of nanoparticle drug uptake and efficacy. This approach mimics critical aspects of the tumor microenvironment while having a reproducible and spatially defined format. Interfacial geometry controls the heterogeneity of the confined cells, where subpopulations with stem cell characteristics are predisposed to periphery regions of high stress, with some characteristics that are reminiscent of the invasive niche in vivo.^[^ ^13^, ^14^ ^]^ This segregation of tumor cell subtypes allows simultaneous assessment of cancer cell and cancer stem‐like cell populations within the patterned aggregate with thundreds of replicates in a single well.

Using B16 melanoma cell lines of varying metastatic potential and polydopamine–fructose–curcumin (PFC3) nanoparticles, we discovered a relationship between tumorigenicity and nanoparticle uptake, with evidence for dynamic changes in membrane deformability in mediating endocytosis. Together, these findings suggest that different subpopulations within a tumor mass may be more susceptible to nanoparticle treatment, thereby providing a new approach for selective therapeutic targeting based on cell state.

## 2 Results and Discussion

### 2.1 Synthesis and Characterization of Cy5‐PFC3 Nanodrug

**Figure 1:**
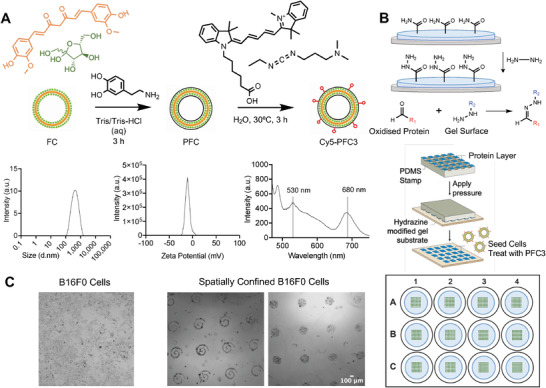
Microcontact printing of fibronectin protein confined cells into biomimetic microtumors for in vitro Cy5‐PFC3 treatment. A) Synthesis scheme for formation of Cy5‐PFC3. Zeta potential (−10.9 mV), size distribution (154.6 d nm, PDI = 0.098), and fluorescence emission profile of Cy5‐PFC3. B) Soft lithography process to covalently immobilize a layer of fibronectin to polyacrylamide hydrogels. Schematic outlining protocol in microcontact printing. Oxidized fibronectin forms stable hydrazone bonds with hydrazine modified gel surface. C) Representative light microscopy images of B16F0 cultured on tissue culture plastic and cells geometrically confined into 250 µm diameter circle and spiral fibronectin micropatterned on nonadherent polyacrylamide gels.

We have chosen polydopamine (PDA) nanoparticles as model drug carriers to be tested in our cell model. PDA nanoparticles and nanocapsules are widely utilized not only because of their biocompatibility, but also due to beneficial properties such as photothermal conversion and the ability to scavenge reactive oxygen species.^[^ ^15^, ^16^ ^]^ Recently, Wong et al. developed a PDA nanocapsule that could be used to entrap curcumin in combination with a range of other hydrophobic drugs.^[^ ^17^, ^18^ ^]^ The attraction of this system does not only lie in the co‐delivery of two drugs for synergistic drug delivery, but also in the high drug loading content. Moreover, the size of the PDA shell of the capsule can influence cellular uptake, drug release, and cytotoxicity.^[^ ^18^ ^]^ We identified here the nanoparticle coined PFC3 nanoparticles (polydopamine–fructose–curcumin, coated for 3 h) from the work by Wong et al. as the most attractive carrier due to the high bioactivity.^[^ ^17^, ^18^ ^]^ PFC3 was first synthesized by mixing fructose and curcumin, which self‐assemble into vesicles by hydrogen bonding (FC in **Figure** **1**A). Addition of Tris buffer and dopamine hydrochloride in an aqueous solution led to the formation of a solid PDA nanocapsule, entrapping curcumin inside (PFC in Figure 1A). Although the PDA nanoparticles are scaffolded around the fluorescent moiety, curcumin, it shares the same emission wavelengths as melanin, which is endogenously produced by melanoma cells. Therefore, the nanoparticles were additionally labeled with Cy5 using the low number of primary amine moieties on the PDA surface, which are utilized to attach the cyanine5 carboxylic acid (Cy5‐COOH) fluorophore through a 1‐ethyl‐3‐(3 dimethylaminopropyl)carbodiimide (EDC) coupling mechanism (Cy5‐PFC3 in Figure 1B,C). The internal curcumin layer acts both as a structural component through hydrogen bonding and *π*–*π* stacking with the fructose, and as a drug component, with studies showing effectiveness against cancer.^[^ ^19^, ^20^ ^]^ The dopamine further allows for fluorescence labeling for tracking in cell studies. The resulting nanoparticles were verified to be monodisperse (PDI < 0.1) through dynamic light scattering analysis. Fluorescence spectroscopy was employed to verify Cy5 incorporation onto the PFC3 nanoparticle through an emission peak at ≈680 nm (Figure 1B). PFC3 has an average diameter of 150 nm and zeta potential of −40 mV prior to Cy5 conjugation, while Cy5 attachment led to an increase in zeta potential to −10 mV.

### 2.2 Cancer Stem‐Like State Influences Uptake of Nanodrug

Cancer cells show broad heterogeneity with cells having variable metastatic potential within a population. Cells cultured in monolayer or organotypic formats will display heterogeneity that varies from plate‐to‐plate and spheroid‐to‐spheroid, thereby making it difficult to relate treatment efficacy to cell subtype. We have developed a method based on soft lithography, where aggregates of cancer cells can be precisely patterned across hydrogels (Figure 1B). The cell laden hydrogel is cast as a thin film onto 18 mm hydroxyl functionalized glass then microcontact printed with ≈100 uniform protein islands, each circle is 240 µm in diameter and spirals are 350 µm from the furthest points, with 600 µm between each feature. Under these culture conditions cells grow in a monolayer which yields a 45 000 µm^2^ microtumor for both the circular and spiral structures. Considering uniform distribution within the cell culture media, diffusion is comparable across the arrayed microtumors.^[^ ^13^ ^]^ This technique has a unique advantage of being able to spatially organize heterogeneity within a pattern, and was shown to direct a cancer stem cell‐like phenotype to perimeter regions.^[^ ^13^ ^]^

To evaluate the effect of Cy5‐PFC3 on cells with varying metastatic potential, we tested the uptake of the particles in our micropatterning system using two related murine melanoma cell lines of the same parental lineage but differing metastatic potential—the B16F0 (low metastatic potential) and B16F10 (high metastatic potential). We prepared 10 kPa hydrazine modified polyacrylamide hydrogels conjugated with fibronectin using soft lithography to pattern different shapes (Figure 1B,C). This modulus was selected as it is representative of mesenchymal tissue stiffness, where tumors are situated.^[^ ^21^ ^]^ First, B16F0 and B16F10 cells were cultured on nonpatterned hydrogels and hydrogels with circle patterns for one and five days. This later timepoint precedes multilayer formation and provides the greatest heterogeneity between cells at the perimeter and the center.^[^ ^13^ ^]^ At these select culture timepoints, cells were treated with Cy5‐PFC3 for 1 h to allow particle uptake into cells. The diffusion of Cy5‐PFC3 into the polyacrylamide hydrogel was negligible, with no evidence of fluorescence present in the hydrogel.

Significant differences in nanodrug uptake were observed between B16F0 cells cultured at the perimeter compared to B16F0 cells cultured at the center of circle patterns at five days, suggesting cells at the boundary are more amenable to nanodrug uptake. Compared to B16F0 cells on nonpatterned hydrogels, the B16F0 cells cultured in circle shapes had a 2.5‐fold increase of Cy5‐PFC3 uptake. There was no significant difference in Cy5‐PFC3 uptake between cells adherent to nonpatterned and circle patterned hydrogels when using the highly metastatic B16F10 cells. We speculate this could be on account of the more metastatic cells being less susceptible to segregation within the pattern area as observed previously.^[^ ^13^ ^]^

**Figure 2:**
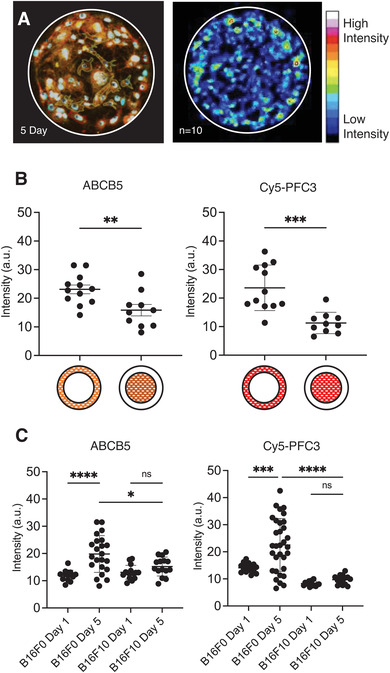
Mechanical cues prime a population of cells with increase stemness which increase uptake of Cy5‐PFC3. A) Representative image of fluorescence intensity quantitation in B16F0 cells (Image J). Cells outlined in red considered “perimeter” and yellow considered “center”. Heatmap of nanoparticle intensity after five days culture (*n* = 10). B) Scatter plot of fluorescence intensity of B16F0 cells localized at the perimeter and the center of a pattern cultured for five days after 1 h treatment with Cy5‐PFC3. Significant difference in uptake between cells in perimeter and center (*p* = 0.0002, *n* = 12) and ABCB5 expression (*p* = 0.0079, *n* = 12). C) Scatter plot of fluorescence intensity of B16F0 and B16F10 cells in spiral patterns cultured on 10 kPa polyacrylamide substrate. Error bars denote ± standard deviation. Significant difference in Cy5‐PFC3 uptake between B16F0 one day and five days (*p* = 0.0005, *n* = 15), B16F0 and B16F10 at five days (*p* < 0.0001, *n* = 15) and ABCB5 expression between B16F0 one day and five day (*p* < 0.0001, *n* = 15), B16F0 and B16F10 at five days (*p* < 0.0174, *n* = 20).

Since convex perimeter geometric cues were previously shown to induce stem fraction in melanoma,^[^ ^13^, ^14^ ^]^ we evaluated correspondence of nanodrug uptake with expression of molecular stemness markers. B16F0 cells at the perimeter of circle patterns had a statistically significant increase in uptake compared to cells at the center after five days of culture. This was not observed when the cells were cultured for one day, with uptake displaying no statistically significant difference between the perimeter and center, suggesting the difference in uptake is related to the gradual change in cell heterogeneity. Consistent with the trend in nanoparticle uptake, immunofluorescence staining of cells at the perimeter showed 1.5‐fold higher expression of the stemness marker ABCB5 compared to cells in the center. To further understand if the perimeter stress in the microtumors is influencing both stem fraction and nanoparticle uptake, B16F0 and B16F10 cells were cultured on a high boundary spiral pattern for one and five days. This geometry was shown previously to enrich stem fraction across the patterned aggregate from <1% to ≈15%.^[^ ^13^ ^]^ Cy5‐PFC3 nanoparticles were added to the cultures at day one and day five for 1 h before fixation and immunofluorescence staining. The results showed B16F0 cells grown over five days had the highest nanoparticle uptake, with a 2‐fold increase on average compared to cells confined for one day. This correlated with ABCB5 expression, which increased ≈2‐fold over five days in cells at the perimeter (**Figure** **2**). Similar to the circle patterns, the B16F10 cells did not show a significant increase in ABCB5 expression or nanoparticle uptake over five days.

**Figure 3:**
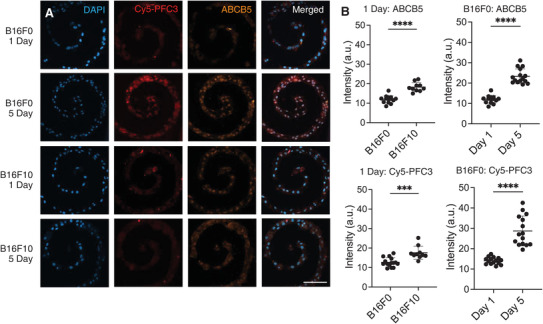
Shape induced and innate cellular ABCB5 expression increases nanoparticle uptake in murine melanoma cells (Scale bar = 100 µm). A) Representative fluorescence images of a B16F0 one day culture, B16F0 five day culture, B16F10 one day culture, and B16F10 five day culture. Fluorescence channels present Cy5‐PFC3 nanoparticle (Red), ABCB5 putative cancer stem cell marker (Alexa Fluor 555, Orange) and cancer cell nuclei (DAPI) on spiral patterns. B) Quantification of mean Cy5‐PFC3 and ABCB5 stem cell marker intensity in B16F0 (*n* = 10) and B16F10 (*n* = 10) cells proliferated under spiral geometry for one day or five days with standard deviation shown with error bars. ****: *P* ≤ 0.0001, ***: *P* ≤ 0.001

To test whether this relationship between interfacial curvature, stem fraction and nanoparticle uptake was not a cell‐type specific result, we also cultured A375 human melanoma cells of varying metastatic potential (Figure S1, Supporting Information). The metastatic cell line A375‐MA.1 show elevated stem markers CD271 but not ABCB5, with a slight increase in nanoparticle uptake compared to the parent line A375‐P (Figure S2, Supporting Information). After culture on the spiral patterned hydrogels for five days we see a significant increase in stem fraction for the A375‐P cells as determined by immunostaining for ABCB5 and CD271, suggesting stem fraction can be primed in human cells at regions of curvature. Similar to our study with B16 cells (**Figure** **3**), this enhancement in stem fraction also corresponds to a significant increase in nanoparticle uptake (Figure S2 **C**, Supporting Information). Therefore, the relationship between “stemness” and nanoparticle uptake is not restricted to the B16 cells but is also observed in human cells of varying metastatic potential.

This result suggests that nanoparticle uptake increases in cells with higher expression of markers associated with cancer stem cell phenotypes. The stem fraction in melanoma ranges from 1.6% to 20.4% and is regarded as a key contributor to growth, metastasis, and drug resistance.^[^ ^22^ ^]^ This subpopulation is capable of self‐renewal and differentiation, giving them the potential for indefinite proliferation, and are also thought to show resistance to chemotherapy and radiotherapy, thereby contributing to recurrence.^[^ ^23^, ^24^ ^]^ As a result, the potential for nanoparticles to localize toward the cancer stem cell population has profound clinical implications, with scope for selective therapeutic targeting of stem fraction.

### 2.3 Enhanced Uptake Relates to Membrane Deformability

**Figure 4:**
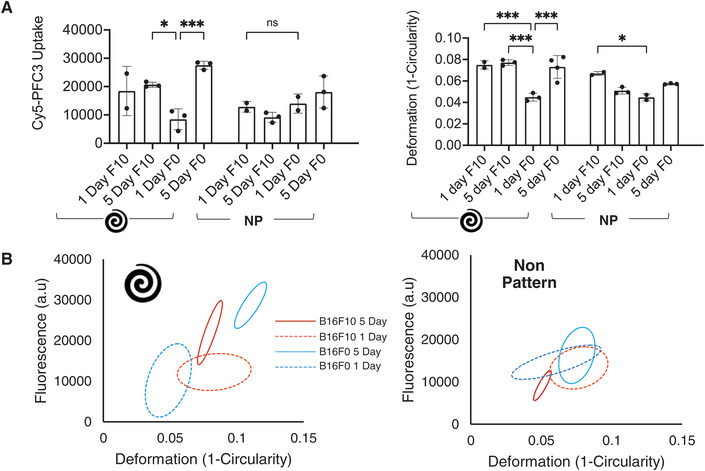
Real time deformation cytometry paired with flow cytometry for simultaneous analysis of B16F0 and B16F10 melanoma cells of membrane deformability and Cy5‐PFC3 uptake. A) Quantification of Cy5‐PFC3 uptake (left) and deformation (right) of B16F0 and B16F10 cells cultured for either one or five days in spiral geometries or nonpatterns, treated with Cy5‐PFC3 for 1 h. Error bars denote ± standard deviation ***: *P* ≤ 0.001, *: *P* ≤ 0.05, ns: *P* ≥ 0.05. B) Correlation between real time deformation and fluorescence of B16F10 and B16F0 cells cultured in spirals or nonpatterns for one or five days, treated with Cy5‐PFC3 for 1 h. Ellipse denotes 0.95 confidence interval.

Nanoparticles are taken up by cells through different mechanisms at the cell membrane, involving numerous proteins whose composition changes depending on particle size, shape, and charge.^[^ ^25^ ^]^ This process of endocytosis is ultimately governed by physical differences in membrane properties that vary with cell type and state.^[^ ^26^ ^]^ Based on the fluidity of the membrane and its protein and receptor composition, cells change the way they interact with extracellular components.^[^ ^27^, ^28^ ^]^ As differences were observed in nanoparticle uptake as a function of heterogeneity in our microconfined melanoma aggregates, we continued to explore whether this corresponded to differences in the physical properties of the cell membrane. We reasoned that probing membrane mechanics across our different cell populations could reveal differences that correspond to stemness and enhanced uptake. B16F0 and B16F10 cells were grown either on spiral, nonpatterned, or plastic surfaces for one or five days and treated with Cy5‐PFC3 for 1 h. Trypsinization was performed on the micropatterned islands to form a single cell suspension, which was analyzed through real‐time cell deformation cytometry. The deformation findings demonstrated that after one day, B16F10 cells showed greater deformation than B16F0 cells irrespective of culture condition; however, after five days, there was a significant increase in deformation for B16F0 cells cultured in spiral patterns. The results further showed a positive correlation between cell deformation and Cy5‐PFC3 uptake (**Figure** **4**). There was a 1.5‐fold increase in cellular deformation and 2.5‐fold increase in Cy5‐PFC3 uptake for B16F0 cells cultured in spiral geometries for five days compared to those cultured for one day. There was no significant difference between any of the cell types cultured on nonpatterned hydrogels with respect to nanoparticle uptake. However, the B16F10 cells cultured for one day on nonpatterned hydrogels demonstrated slightly higher deformability compared to B16F0 cells.

These cell deformation results provide evidence that culture on the spiral geometry enriches a cell subpopulation with increased membrane deformation, coinciding with stem cell‐like characteristics. Additionally, to understand the relationship between nanoparticle uptake and deformation, we calculated the fluorescence (uptake) as a function of deformation for each condition (Table S1 and Figure S3, Supporting Information). The change in this parameter due to microconfinement (spiral – nonpattern) can be presented as a “uptake factor” with B16F0 5 day > B16F10 5 day > B16F10 1 day > B16F0 1 day. The uptake factor corresponds with cancer “stemness” supporting our hypothesis that nanoparticle drugs may be well suited for targeting stem fraction in cancer. Cellular deformation is recognized as a key characteristic in metastatic cell states as they hold the capacity to migrate in confinement.^[^ ^29^ ^]^ Previous studies have shown that cancer subpopulations which are softer and have high motility are more likely to metastasize.^[^ ^30^, ^31^ ^]^ This can be linked to membrane tension, where motile cells experience decreased membrane packing, which can impact both cell invasion and transport of objects across the lipid bilayer.^[^ ^32^ ^]^

To further test our hypothesis that stem fraction shows variable membrane tension, which corresponds to increased nanoparticle uptake, we performed fluorescent lifetime intensity microscopy (FLIM) using Flipper‐TR dye, which provides a measure of membrane tension in adherent cells. FLIM imaging of the B16F0 cells at day one and day five indicates decreased membrane tension over culture time corresponding to increased stem fraction, with a clear radial analysis indicating lower membrane tension in cells adjacent to the curved boundary (Figure S4, Supporting Information). These results correspond well with the deformability measurements. The variation in membrane tension on account of metastatic potential may account for the increased Cy5‐PFC3 uptake in cells with the cancer stem cell phenotype, as they feature lower membrane order which could increase nanoparticle transport across the bilayer.^[^ ^32^ ^]^

### 2.4 Change in Microtumor Area and Cellular Detachment Induced by Cy5‐PFC3

Having demonstrated correspondence of uptake and membrane deformation, we asked whether the stemness‐uptake relationship would provide an advantage for targeting these populations with nanodrugs. To investigate if higher stemness corresponds with increased drug responsiveness, B16F0 and B16F10 cells were seeded on micropatterned circles, spirals, and nonpatterned hydrogels across 10 kPa stiffness tuned polyacrylamide gels for either one or five days. The live cells were then stained with Hoechst to identify nuclei and treated with Cy5‐PFC3 (80 µg mL^−1^, 1 mL). The cells were imaged using widefield microscopy every 15 min over 24 hours to visualize cell detachment and changes in cellular morphology on account of treatment with the drug curcumin from the PCF3 nanoparticles. The response of the cells over an extended period to a toxic dose of nanoparticles (IC50 over 24 h for B16F0 and B16F10 being 12 and 15 *µ* m, respectively) were compared and a change in number of nuclei and area of the microtumor were determined as a measure of cell death over time.

**Figure 5:**
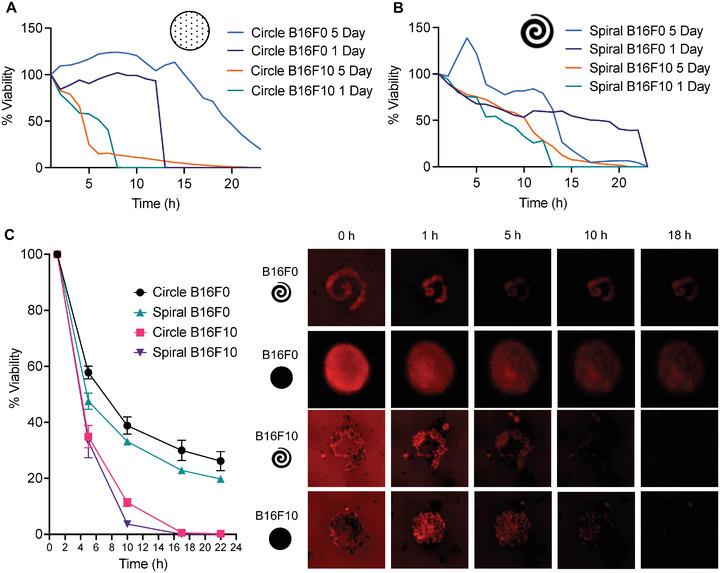
Time series measurement of microtumor area shows increased death in more metastatic cells. All data normalized to initial timepoints. A) Measurement of microtumor area for B16F0 and B16F10 melanoma cells patterned on circle geometries for 24 h after Cy5‐PFC3 addition. Cells were patterned for either one or five days prior to Cy5‐PFC3 addition. B) Measurement of microtumor area for B16F0 and B16F10 melanoma cells patterned on spiral geometries for 24 h after Cy5‐PFC3 addition. Cells were patterned for either one or five days prior to Cy5‐PFC3 addition. C) Quantification of average Cy5‐PFC3 intensity in patterned region. Measurements taken at 1, 5, 10, 18, and 22 h. Representative images of Cy5‐PFC3 visualized through Cy5 label (Red).

When under geometric confinement for a short period of time, where the cell population is homogenous, the more metastatic B16F10 cells had increased susceptibility to Cy5‐PFC3 treatment, detaching from the surface 18% faster compared to the less metastatic B16F0 cells (**Figure** **5**; Figure S5, Supporting Information). Similarly, on circle patterns the B16F10 cells detached much more readily compared to the B16F0 cells, irrespective of whether they were treated at day one or day five. However, culture on spiral patterned gels had a marked influence on drug susceptibility, leading to an increased rate of detachment for all cell types compared to non‐patterned and circle patterned gels. The rate of detachment from spiral patterns was comparable for B16F10 cells after nanodrug treatment at day one and day five. However, the B16F0 cells detached 61% faster from spiral patterned gels after nanodrug treatment at day five compared to day one. This result is consistent with the observation that B16F0 cells are sensitive to geometric features, leading to increased stem fraction at day five coinciding with increased nanodrug uptake. These results were confirmed using live/dead staining which demonstrates the same trend in decreased viability across these populations (Figure S6, Supporting Information). Overall, this result suggests that cells, which exhibit a greater stem fraction either natively or induced through patterning, have increased susceptibility to nanodrug uptake, corresponding to loss of viability and detachment from the substrate. The detached cells for all conditions were collected and reseeded into a fresh tissue culture plate and monitored for seven days with regular media changes to assess viability after drug treatment. The cells released were either dead or remained viable but unable to proliferate, confirming the validity of this assay to quantify drug‐induced cell death. Since there were comparable numbers of cells released from each condition, we believe the released cells undergo apoptosis or drug‐induced senescence rather than anoikis related to varying metastatic potential.

Here we have investigated Cy5‐PFC3 nanoparticle uptake in metastatic melanoma cells with the aim of identifying differences in localization on a population of cells influenced by a defined geometry creating a heterogeneous population. While much is known about cancer cell plasticity and tumors harboring different cell subpopulations, the effect of nanoparticle drugs on heterogeneous cell types has not been explored.^[^ ^33^ ^]^ Here we used a simple and reproducible 2D hydrogel model to recapitulate heterogeneous features of tumor tissue to study nanoparticle uptake.

Melanoma cells of high metastatic potency are very dangerous and difficult to target and were therefore selected to test the model. After treatment with Cy5‐PFC3 nanoparticles, there was increased uptake in cells which exhibited higher expression of stem cell markers. This corresponded to viability where prolonged treatment led to faster detachment from the substrates. Compared to the current 2D gold standard of plastic tissue culture plates, which produces a homogenous monolayer of cells, the reproducible positioning of stem cell‐like cells allowed comparison of nanoparticle uptake across cell subpopulations within and across microtumors.

To understand the differences between cell types with respect to nanoparticle uptake, we released cells from a high stem fraction condition (spiral patterned gel), a medium stem fraction condition (circle patterned gel), and a low stem fraction condition (nonpatterned gel) for further analysis. Deformation cytometry, a technique that measures the stiffness and deformability of cells, demonstrated a 1.5‐fold increase in cellular deformation and 2.5‐fold increase in Cy5‐PFC3 uptake for high stem fraction conditions compared to low stem fraction conditions. This result suggests a relationship between stemness, membrane tension, and nanoparticle uptake. Previous studies have suggested cells with increased metastatic potential dysregulate and modulate signaling through increased endocytosis, altering their membrane composition.^[^ ^34^ ^]^ It has also been suggested cells can actively mechanoadapt their membrane tension during nanoparticle uptake, although the reason for this remains unclear.^[^ ^35^ ^]^ Future work can extend this platform to include stromal and fibrotic elements with cellular cocultures to further recapitulate the tumor microenvironment. For instance, we recently demonstrated the use of microtumor arrays as a tool to study cancer associated fibroblast interactions with pancreatic cancer, where the fibroblasts adopted precise spatial locations on account of interfacial stress. This platform could prove useful to study how stromal elements may influence nanoparticle uptake. Our results demonstrate that increased cancer cell stemness corresponds to increased membrane deformability, which makes these cells more susceptible to nanoparticle uptake. This finding suggests a novel route in the development of anticancer drugs‐targeting stem fraction with nanoparticle therapeutics—which could aid treatments targeting this elusive subpopulation that is involved in recurrence, resistance, and metastasis.

## 3 Conclusions

In this work, we have shown the application of polyacrylamide hydrogels micropatterned with geometric protein islands to create microtumor arrays for the study of nanodrugs. We discovered a relationship between stem fraction, membrane deformation, and nanoparticle uptake, and demonstrated that extended nanoparticle treatment induced cell detachment and death. These results together suggest nanomedicine may be better predisposed for targeting stem fraction in patients, which may prove useful in treatment of advanced disease to supplement standard of care.

## 4 Experimental Section

### Synthesis of PFC3

Fructose (100 mg, 10 mg mL^−1^) was dissolved in Milli‐Q water (10 mL) and sonicated for 10 min. Curcumin in DMSO (200 µL, 4 mg mL^−1^) was added to the fructose solution dropwise and the solution was gently homogenized. Aqueous dopamine hydrochloride (100 µL, 10 mg mL^−1^) then Tris‐hydrochloride (100 µL, 6 mg mL^−1^) was added and the solution was polymerized in the dark for 3 h. The resulting solution was dialyzed against Milli‐Q water for 24 h.

### Synthesis of Cy5‐PFC3

N‐(3‐Dimethylaminopropyl)‐*N*′‐ethylcarbodiimide hydrochloride (EDC) (25 µL, 1 mg mL^−1^) was added to the nanoparticle solution (10 mL) at 30 °C for 1 h. Cyanine5 carboxylic acid (25 µL, 1 mg mL^−1^) was added to the mixture and coupled for 2 h at 30 °C with constant gentle mixing. The solution was centrifuged at 15 000 rpm and the Cy5‐PFC3 pellet was redispersed in Milli‐Q water and washed three times.

### Dynamic Light Scattering and Fluorometer

Particle size and zeta potential was measured using DLS on a Zetasizer Nano ZS (Malvern Panalytical). The aqueous sample was measured with a noninvasive back scatter detector and short measurement distance to reduce scattering and thereby error in the measurement. All samples were measured at 25 °C with no equilibration time. Cy5 dye attachment was confirmed using Cary Eclipse Fluorescence Spectrophotometer. The aqueous solution was sampled with excitation wavelength at 450 nm and the excitation and emission slit at 10 nm. Fluorescence emission was collected between 470 and 800 nm.

### Polyacrylamide Hydrogels

Polyacrylamide hydrogels with a 10 kPa stiffness was fabricated on 18 mm glass coverslips following established protocols. To summarize, the circle glass coverslips were hydroxylated through sonication in ethanol for 15 min, then sonicated in Milli‐Q water for 15 min. The activated coverslips were aminosilanized through treatment with 0.5% 3‐aminopropyltriethoxysilane, the functionalized with 0.5% glutaraldehyde for 30 min, with three washes between each treatment. The coverslips were dried using a stream of nitrogen. To polymerize and formulate the hydrogel, 40% acrylamide, and 2% bis‐acrylamide stock solutions were mixed and degassed with argon to the desired stiffness. The reaction was initiated by ammonium persulfate (5 µL) and catalyzed by tetramethylethylenediamine (0.5 µL) when added to the acrylamide/bis‐acrylamide solution (500 µL). The mixture (20 µL) was pipetted onto a hydrophobically treated glass slide and the modified surface of the coverslip was placed on the droplet, spreading the prepolymerized solution. After 25 min of polymerization, the hydrogel coated coverslip was gently detached from the glass slide. These polyacrylamide hydrogels were stored in distilled water at 4 °C until use.

### Hydrazine‐Modified Polyacrylamide Hydrogels

Polyacrylamide hydrogels were functionalized with hydrazine monohydrate for 30 min and rinsed with Milli‐Q water three times. The gels were treated with 1% acetic acid for 1 h and rinsed three times with Milli‐Q water. The hydrazine modified hydrogels were left overnight at 4 °C in Milli‐Q water before use.

### Polydimethylsiloxane Stamps

Polydimethylsiloxane (PDMS) stamps were fabricated though polymerization on a silicon master patterned with photoresist, created using ultraviolet photolithography through a laser printed mask. An even layer of a 10:1 mixture of PDMS base to crosslinker was poured onto the silicon master and placed into a degassing chamber until bubbles were removed. The PDMS was cured at 60 °C for 12 h, then detached from the master. The resulting stamps were sonicated in ethanol for 15 min to sterilize before use.

### Micropatterned Hydrogels

PDMS stamps were cleaned by sonication in ethanol then water for 15 min. Fibronectin dispersed in PBS was oxidized with sodium periodate for 45 min, then pooled onto the patterned surface of the stamps for 30 min. The stamp surface was gently dried with a stream of nitrogen gas to reveal a white fibronectin protein layer on the stamping surface. The oxidized fibronectin was transferred through contact onto the hydrazine modified polyacrylamide hydrogel applying light pressure to ensure complete transfer. The fibronectin patterned hydrogels were stored in Milli‐Q water at 4 °C until use.

### Cell Culture

B16F0 and B16F10 murine melanoma cells and A375‐P and A375‐MA.1 human melanoma cells were thawed from cryopreservation (5% DMSO, 95% culture medium) and cultured in high glucose Dulbecco's modified Eagle's medium (DMEM, 4.5 g/L d‐glucose, l‐glutamine, 0.11 g L^−1^ sodium pyruvate) supplemented with 10% fetal bovine serum (FBS) and 1% penicillin–streptomycin (P/S). Culture medium was replaced every 2 days and the cells were passaged at 90% confluency using 0.25% trypsin‐EDTA, centrifuging at 125 × g for 5 min for B16F0 and B16F10 and 0.05% trypsin‐EDTA, centrifuging at 300 × g for 3 min for A375‐P and A375‐MA.1. All cell experiments were performed at low passages of less than 30. B16F0 and B16F10 cells were seeded at either 10 000 cells for 1 day or 1000 cells for 5 days on patterned hydrogels. A375‐P and A375‐MA.1 were seeded at 45 000 for 1 day and 8000 for 5 days on patterned hydrogels, selected to achieve confluency at the chosen timepoints.

### Optical Microscopy

Sterile cell cultures were imaged with an Olympus CKX52 optical light microscope under the 4× and 10× objective.

### Cy5‐PFC3 Treatment

Patterned hydrogels with cells were treated with Cy5‐PFC3 either 1 day or 5 days after initial cell seeding. Cy5‐PFC3 (80 µg mL^−1^) was centrifuged to remove the aqueous supernatant and redispersed into culture medium. The solution was sterilized by either 0.45 µL sterile filtration or as a dispersed layer under UV light for 15 min. 1 mL of the nanoparticle suspension was added onto each hydrogel. The cells received treatment for 1 h in uptake studies and 24 h in live cell studies. Cells in uptake studies were fixed with 4% paraformaldehyde before imaging.

### Immunofluorescence

Cells attached on gel treated coverslips were fixed with 4% paraformaldehyde for 20 min, then permeabilized with 0.1% Triton‐X in PBS for 30 min. 1% BSA in PBS was added for 15 min to block nonspecific binding of the antibodies. Gels were then incubated with the primary antibody (rabbit polyclonal anti‐ABCB5, Novus Biologicals) diluted at 1:500 in 1% BSA in PBS for 1 h at room temperature. Secondary antibody labeling (goat anti‐rabbit Alexa Fluor 555, Sigma‐Aldrich) was performed in 1% BSA in PBS at 1:200 dilution for 1 h at room temperature in the dark. The cells were gently washed twice with PBS between each reagent addition. The coverslips were stored in PBS at 4 °C in the dark until ready for mounting and imaging. The patterned gel coverslips were mounted to glass slides using Fluoroshield with DAPI mounting media (Sigma‐Aldrich).

### Fluorescent Lifetime Measurement

B16F0 cells were grown on fibronectin coated 250 µm circle patterns on a 10 kPa polyacrylamide substrate in glass bottom dishes for 1 day and 5 day. On the day of the experiment, the cell culture medium was replaced with a staining solution (2 µm Flipper‐TR in complete culture medium) and placed at 37 °C in a humidified atmosphere containing 5% CO_2_. After 30 min, the staining solution was removed, cells were washed once with prewarmed PBS and imaging was performed in complete culture medium.

FLIM imaging was performed using a Picoquant Microtime 200 STED confocal microscope as described previously.^[^ ^37^ ^]^ Excitation was performed using a pulsed 485 nm laser Diode (PicoQuant, LDH‐D‐C‐485) operating at 20 MHz, and emission signal was collected through a bandpass 600/50 nm filter using a gated PMA hybrid 40 detector and a TimeHarp 260 NANO TCSPC board (PicoQuant). SymPhoTime 64 software (PicoQuant) was then used to process and analyze the data. A customized Matlab script (Matlab) was used to extract average lifetime at each radius from the center to the edges of the circle patterns and data is shown as mean ± SEM from 5 to 6 individual patterns.

### Confocal Microscopy

Fixed cell images were acquired using laser scanning confocal microscopy on a Zeiss LSM 800. Images were captured with a 20×/0.8 objective. DAPI, Alexa Fluor 555 and Cy5 were excited with 405, 561, and 640 nm laser lines, respectively.

Representative images were processed and analyzed using ImageJ (NIH, USA).

### Widefield Microscopy

Live cell images were acquired on the Zeiss Celldiscoverer 7 Widefield Microscope under a 5×/0.35 objective lens and 2× tube lens giving an effective magnification of 10×. Images were acquired every 15 min for 24 h. The cells were maintained at 37 °C and 5% CO_2_ in high glucose DMEM media with 10% FBS, and 1% P/S for the duration of imaging. Hoechst and the Cy5 nanoparticle were excited by 385 and 625 nm LED lines, respectively.

### Heatmap Data Analysis

ImageJ (NIH, USA) software was used to analyze immunofluorescence images to generate heatmaps. Raw images were imported, and background subtracted. Ten (*n* = 10) patterns were stacked and translated to align all stacks. Z‐Project of the stack was converted to 16 colors lookup table.

### Real‐Time Deformability Cytometry

Membrane deformability was measured on the AcCellerator Deformation Cytometer (Zellmechanick Dresden). The width of the microfluidics channel used was 30 µm, with a sample flow rate of 0.040 *µ*L s^−1^ and a sheath flow rate of 0.120 *µ*L s^−1^. CellCarrier solution (Zellmechanick Dresden) was used as the sheath fluid and for sample resuspension and the mean chip temperature was 23.8 °C. The 647 nm laser line was used to determine the fluorescence intensity of the Cy5‐PFC3 treated cells.

### Cell Replating

B16F0 and B16F10 cells cultured on circle and spiral patterned surfaces for 1 or 5 days were treated with 80 µg mL^−1^ Cy5‐PFC3. At the 1 and 3 h timepoints, cells which had detached were centrifuged at 125 × *g* for 5 min and resuspended in complete tissue culture media (10% FBS, 1% P/S in high glucose DMEM). The cells were then placed into a well in a 12‐well plate and observed for 7 days.

### Statistics

GraphPad Prism 8.0.2 was used for statistical analysis. Sample size was a minimum of ten (*n* = 10) for fixed circle and spiral patterns and three for live cell imaging (*n* > 3). Data are shown as means with ± standard deviation (SD). Representative images were selected for ABCB5 expression and nanoparticle uptake visualization. Data were tested for normality using a Shapiro‐Wilk test. An ordinary one‐way ANOVA for three data sets or more was performed with significance determined from a Tukey's multiple comparisons test. Unpaired *t*‐test was performed when comparing two data sets. A *p*‐value of <0.05 was considered statistically significant.

## Conflict of Interest

The authors declare no conflict of interest.

## Supporting information

Supporting Information

## Data Availability

The data that support the findings of this study are available from the corresponding author upon reasonable request.
