# Supplementary material for: Hydrogel Microtumor Arrays to Evaluate Nanotherapeutics
Source: Adv Healthc Mater. 2022 Nov 28;12(14):2201696. doi: 10.1002/adhm.202201696 (PMC11323127; doi:10.1002/adhm.202201696)
Supplement: Supplementary file 1 — Supporting Information [file ADHM-12-2201696-s001.pdf]

ADVANCED HEALTHCARE MATERIALS

Supporting Information

for *Adv. Healthcare Mater.*, DOI 10.1002/adhm.202201696

Hydrogel Microtumor Arrays to Evaluate Nanotherapeutics

Yiling Liu, Stephanie Nemec, Chantal Kopecky, Martina H. Stenzel and Kristopher A. Kilian**

Supporting Information

Hydrogel microtumor arrays to evaluate nanotherapeutics

Yiling Liu, Stephanie Nemec, Chantal Kopecky, Martina Stenzel*, Kristopher A. Kilian*

School of Chemistry, Australian Centre for NanoMedicine, The University of New South Wales, Sydney 2052, New South Wales, Australia

School of Materials Science and Engineering, The University of New South Wales, Sydney 2052, New South Wales, Australia

*k.kilian@unsw.edu.au, m.stenzel@unsw.edu.au

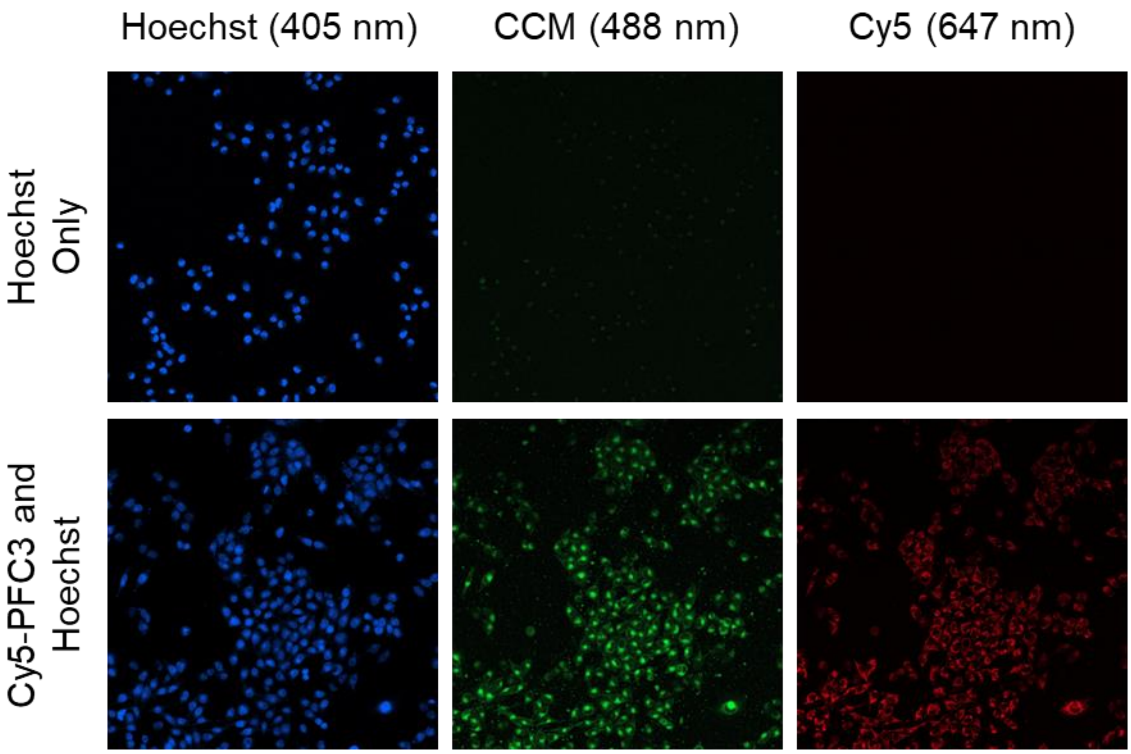

Figure S1. A375-P human melanoma proliferated on glass bottom plates and treated with Cy5-PFC3 for 1 h. Fluorescence channels present Cy5-PFC3 nanoparticle (Red), Curcumin (Green) and cancer cell nuclei (Hoechst).

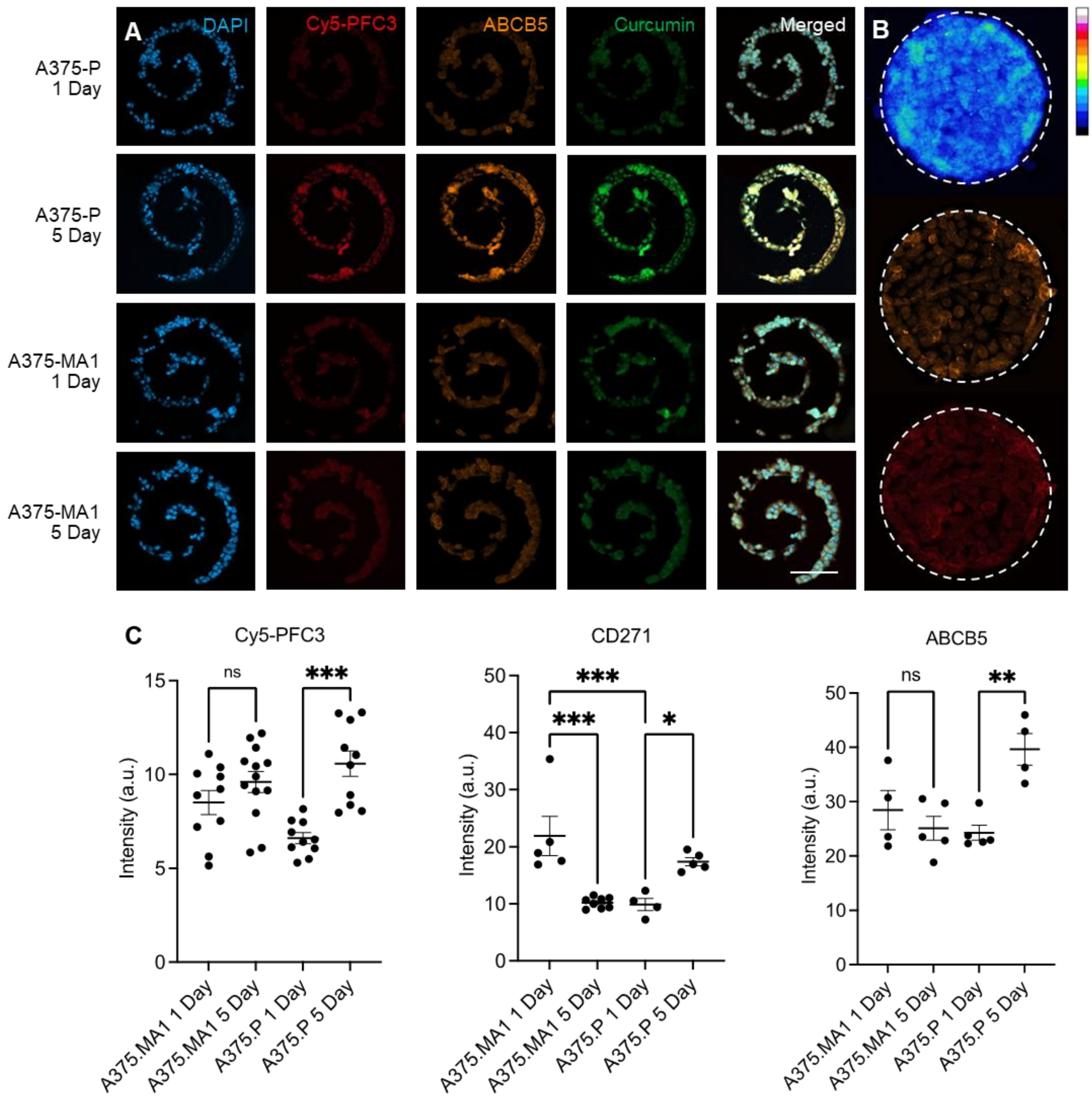

Figure S2. **(A)** Representative fluorescence images of a A375-P one day culture, A375-P five-day culture, A375-MA1 one day culture, and A375-MA1 five day culture. Fluorescence channels present Cy5-PFC3 nanoparticle (Red), ABCB5 putative cancer stem cell marker (Alexa Fluor 555, Orange), curcumin (Green) and cell nuclei (DAPI) on spiral patterns. **(B)** Representative image of fluorescence intensity quantitation of Cy5 intensity in A375-P cells after 5 days of culture, followed by treatment with Cy5-PFC3 for 1h. Colour bar indicates minimum (bottom, dark blue) to maximum (top, white) intensity. **(C)** Quantification of mean ABCB5 and CD271 stem cell marker and Cy5-PFC3 intensity in A375-MA1 (n = 10) and A375-P (n = 10) cells proliferated in spiral geometry for one day or five days with standard deviation shown with error bars. ***: P ≤0.001. **: P ≤0.01, *: P ≤ 0.05

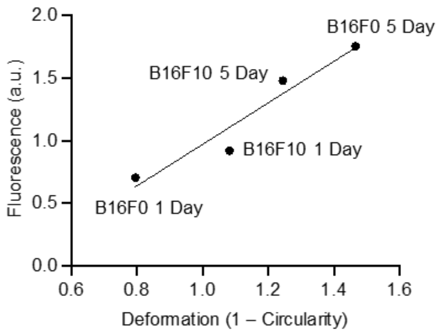

Avg Spiral/Avg Non Pattern	**Deformation**	**Fluorescence**
**B16F0 1 Day**	0.79537736	0.70517649
**B16F0 5 Day**	1.46596020	1.75495545
**B16F10 1 Day**	1.08213657	0.92123682
**B16F10 5 Day**	1.24417662	1.48023852

Table S1. Correlation between real time deformation and fluorescence of B16F10 and B16F0 cells cultured in spirals or non-patterns for 1 or 5 days, treated with Cy5-PFC3 for 1 h. Deformation and fluorescence computed as fold-increase compared to non-pattern baseline.

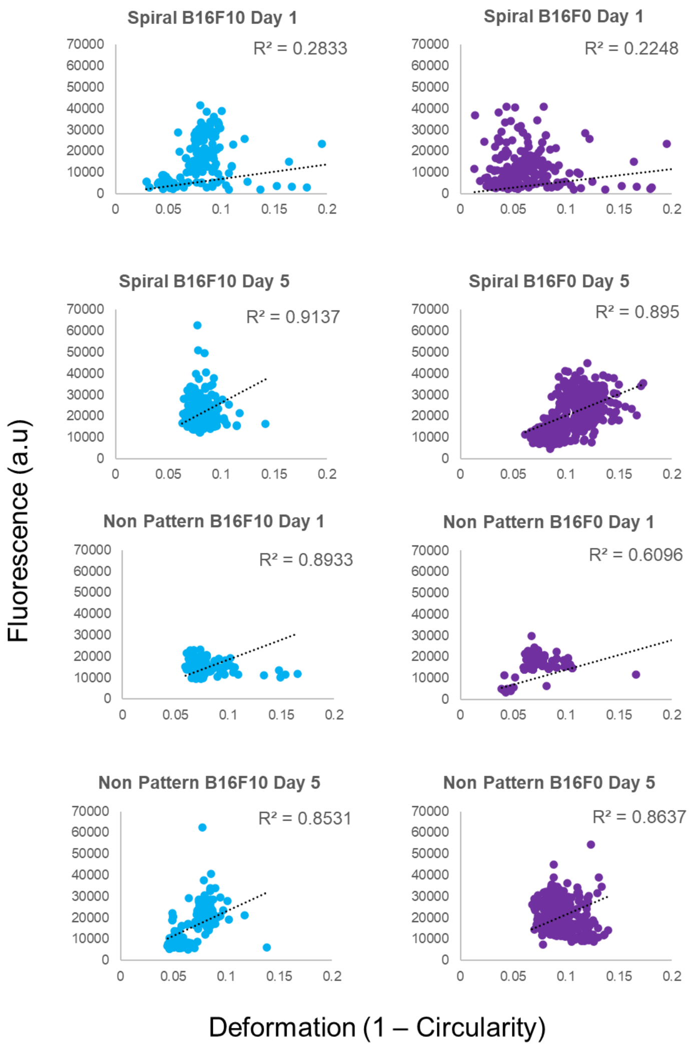

Figure S3. Correlation between real time deformation and fluorescence of B16F10 and B16F0 cells cultured in spirals or non-patterns for 1 or 5 days, treated with Cy5-PFC3 for 1 h.

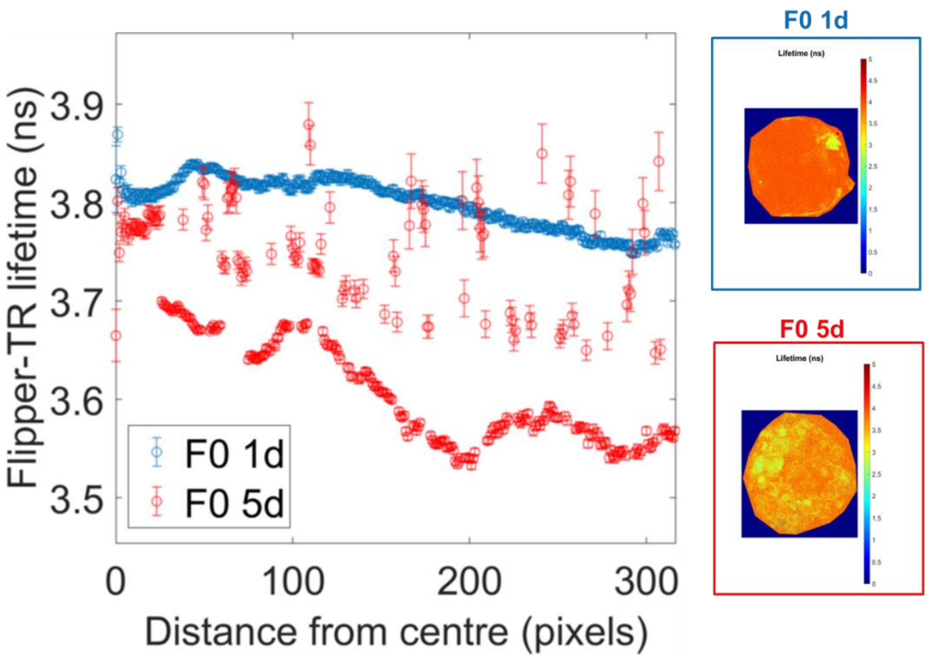

Figure S4. Florescence lifetime imaging of membrane tension probe Flipper-TR indicating a shorter lifetime corresponding to a lower membrane tension in cells after 5 days and at the pattern boundary.

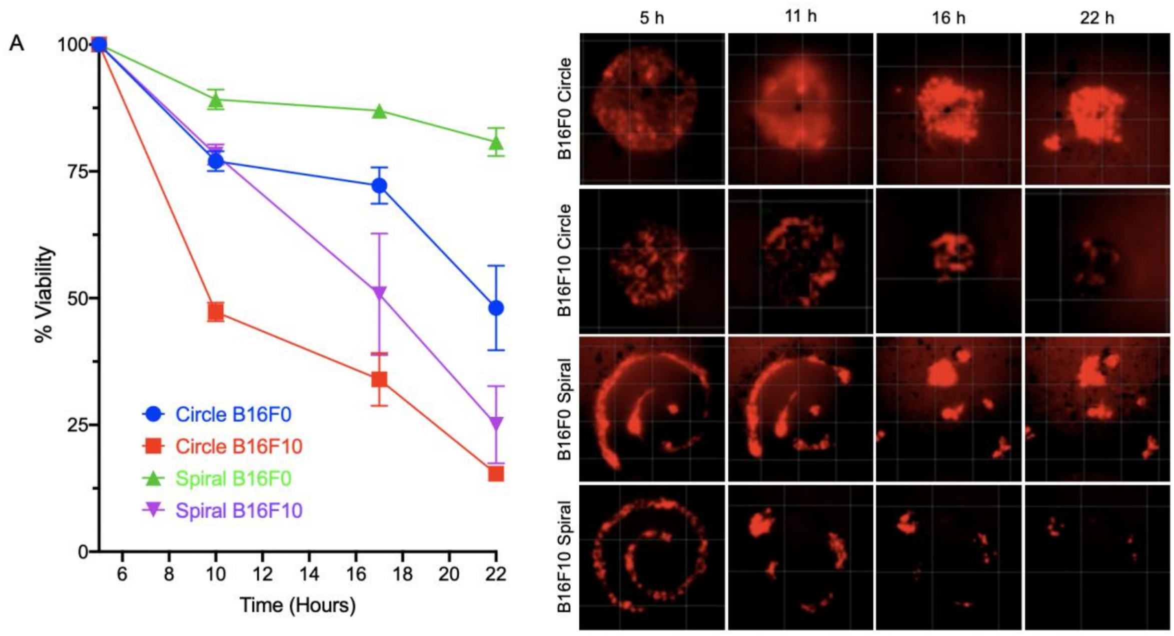

Figure S5. Quantification of cell detachment from micropatterns: initial, mid and final points of 5, 11, 16, 22 h timepoints of B16F0 and B16F10 cells both cultured in spirals and circles. Cy5-PFC3 visualised through Cy5 label (Red).

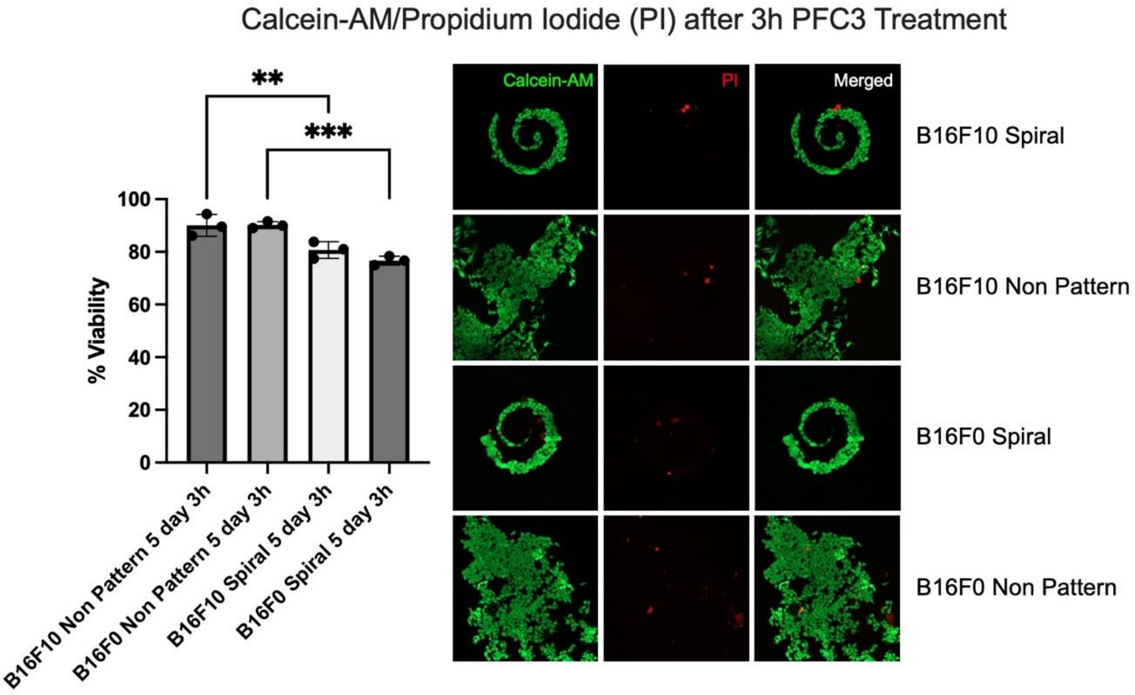

Figure S6. Quantitation of viability in B16F0 (n=3) and B16F10 (n=3) determined by dividing the number of viable cells with the total number of cells and converting to percentage with standard deviation shown with error bars. ***: P ≤0.001. **: P ≤0.01. Representative fluorescence images of a B16F0 and B16F10 at five day of culture, followed by PFC3 treatment for 3h. Fluorescence channels present live cells stained with calein-AM (Green) and dead cells stained with propidium iodine (Red).’
